# Caveolin-1 Scaffolding Domain Peptide Regulates Colon Endothelial Cell Survival through JNK Pathway

**DOI:** 10.1155/2020/6150942

**Published:** 2020-03-06

**Authors:** Kai Fang, Christopher G. Kevil

**Affiliations:** ^1^Inflammatory Bowel Disease Center, Vatche and Tamar Manoukian Division of Digestive Diseases, David Geffen School of Medicine, University of California, Los Angeles, California 90095, USA; ^2^Department of Pathology, Louisiana State University Health Sciences Center, Shreveport, LA 71103, USA

## Abstract

It has been reported that pathological angiogenesis contributes to both experimental colitis and inflammatory bowel disease. Recently, we demonstrated that endothelial caveolin-1 plays a key role in the pathological angiogenesis of dextran sodium sulfate (DSS) colitis. However, the molecular mechanism of caveolin-1 regulation of endothelial function is unknown. In this study, we examined how the antennapedia- (AP-) conjugated caveolin-1 scaffolding domain (AP-Cav) modulates vascular endothelial growth factor- (VEGF-) dependent colon endothelial cell angiogenic responses, as seen during colitis. We used mouse colon endothelial cells and found that AP-Cav significantly inhibited VEGF-mediated bromodeoxyuridine (BrdU) incorporation into colon microvascular endothelial cells. AP-Cav significantly blunted VEGF-dependent extracellular signal-regulated kinase 1/2 (ERK 1/2) phosphorylation at 10 minutes and 2 hours after stimulation, compared with the AP control peptide. AP-Cav + VEGF-A treatment also significantly increased c-Jun N-terminal kinase (JNK) phosphorylation at 2 hours. AP-Cav + VEGF-A treatment significantly downregulated retinoblastoma (Rb) protein levels, upregulated cleaved caspase-3 protein levels at 4 hours, and induced apoptosis. Thus, our study suggests that disruption of endothelial caveolin-1 function via the AP-Cav diverts VEGF signaling responses away from endothelial cell proliferation and toward apoptosis through the inhibition of mitogen-activated protein (MAP) kinase signaling and the induction of JNK-associated apoptosis.

## 1. Introduction

Angiogenesis, the formation of new blood vessels from the existing vascular system, is now a target for inflammation-related diseases. Experiments in our lab [1] and others [2] show that inhibiting angiogenesis attenuates experimental colitis. Endothelial cells play an important role in angiogenesis. Caveolin-1 is a marker protein for the endothelial cell membrane domain caveolae, which are invaginations in the plasma membrane [3]. Many signaling molecules, such as VEGF receptor, EGF receptor, platelet-derived growth factor (PDGF) receptor, and ERK 1/2, are localized in the caveolae^,^ and play a crucial role in angiogenesis [4, 5].

The caveolin-1 scaffolding domain (CSD) peptide reacts with molecules that have the motif ФXXXXФXXФ or ФXXXXФXXФ, where Ф are aromatic acids. AP-Cav is the cell-permeable caveolin-1 scaffolding domain peptide conjugated with the cell-permeable peptide antennapedia which has a molecular weight of 4746.6 Da. AP-Cav is also known as Cavtratin and Pen-C1-SD [6]. Treatment of DSS colitis mice with the AP-Cav peptide resulted in reduced disease activity index, reduced histopathology score, and decreased angiogenesis, suggesting that AP-Cav has the potential to treat colitis [1]. Angiogenesis involves endothelial cell proliferation, migration, and differentiation. The exact molecular mechanism by which the AP-Cav peptide affects colon endothelial cell proliferation is not clear. To see the effects of AP-Cav on proliferation, we used mouse colon endothelial cells as a model to study this molecular mechanism *in vitro*.

## 2. Materials and Methods

### 2.1. Materials

ERK 1/2 antibody was purchased from Cell Signaling. Mouse recombinant VEGF164 (Cat#676474) was purchased from Calbiochem. Mouse monoclonal antibody against retinoblastoma protein (Part#AHO0182) was purchased from Biosource. Rabbit antibody against p27^kip1^ tumor suppression protein (AB3003) was purchased from Millipore. Cleaved form of caspase 3 antibody (Cat#9664) was purchased from Cell Signaling. The AP-Cav sequence used for this study is RQIKIWFQNRRMKWKKDGIWKASFTTFTVTKYWFYR. Phospho-JNK antibody was purchased from Invitrogen (Cat#44682G). JNK inhibitor was purchased from Calbiochem (Cat#420116). AP is the Drosophila Antennapedia Homeo-Domain (43–58) peptide (MW 2246.8 Da) used as a control to AP-Cav. The sequence of AP used in this study is RQIKIWFQNRRMKWKK. DNA fragmentation detection kits were purchased from Calbiochem (Cat#QIA39).

### 2.2. Cell Culture

Colon mucosa was collected from mice with the temperature-sensitive SV40 large T antigen (ImmortoMice). Mucosa was placed into PBS at 4°C, cut into small fragments, digested at 4°C with 0.25% type IV collagenase for 24 hours, and then incubated at 37°C for 1 hour with shaking. Suspensions were passed through a 100 *μ*m diameter pore nylon mesh and then filtered across a 20 *μ*m mesh. The microvessel fragments were collected on the top of the 20 *μ*m mesh with Hank's Balanced Salt Solution (HBSS). Microvessel fragments were cultured in 10 cm dishes using endothelial cell enrichment media, containing MEM D-Valine supplemented with 10% fetal bovine serum, 2 mM L-glutamine, 1% nonessential amino acids, and 1% antibiotic/antimycotic. Primary cell cultures were grown under routine immortalizing culture conditions at 33°C in medium containing 10 U/ml of murine interferon-*γ*, as previously reported [7]. For experiments, cells were seeded into 10 cm dishes and grown to confluency. At time of confluency, the immortalizing medium was replaced with normal medium, and the cells were cultured at 38°C to inactivate large T-antigen expression and immortality for 72 hours.

### 2.3. Proliferation Assay

Mouse colon endothelial cell proliferation was determined by BrdU incorporation using the Calbiochem BrdU cell Proliferation Assay kit (Calbiochem, catalog#219482). Cells were seeded at 1000 cells per well in 96 well plates and cultured for 24 hours. Cells were serum-starved overnight and then pretreated with 7 *μ*M of AP control or AP-Cav peptide for 30 minutes. After peptide pretreatment, cells were stimulated with 50 ng/ml VEGF-A. BrdU working solution was added to each well, and cells were incubated for 4 hours. After the incubation period, the media was discarded and the plate was blotted on paper towels. Cells were then fixed and denatured using fixative/denaturing solution and then incubated at room temperature for 30 minutes. The fixative/denaturing solution was discarded, Anti-BrdU antibody was added to each well, and then the solution was incubated for 1 hour at room temperature. After incubation, the cells were washed 3 times with wash buffer and were incubated with diluted peroxidase-conjugated secondary antibody for 30 minutes at room temperature. After incubation, the cells were washed 3 times with 1× wash buffer, and the amount of antibody staining was detected by adding TMB solution and incubating at room temperature for 5 minutes. The reaction was then terminated using stop solution, and the BrdU incorporation was read on a plate reader at a dual wavelength of 450–540 nm.

### 2.4. Western Blot Analysis

Cell lysates were prepared using RIPA lysis buffer (150 mM NaCl, 1% NP-40, 0.5% deoxycholate, 0.1% SDS, and 50 mM Tris, pH 8.0 with standard protease and phosphatase inhibitors including phenylmethanesulfonylfluoride (PMSF), aprotinin, pepstatin, leupeptin, sodium orthovanadate, and okadaic acid). Total protein levels were measured using the Bio-Rad DC protein assay kit. An equal amount of total protein was loaded onto SDS polyacrylaminade gels and separated by electrophoresis. Proteins were transferred to polyvinylidene difluoride (PVDF) membranes and blocked overnight at 4°C. Membranes were incubated with primary antibody in a 5 ml volume with gentle agitation for 2 hours at room temperature or overnight at 4°C. After being washed 3 times, membranes were incubated with an appropriate HRP-conjugated secondary antibody for 1 hour at room temperature. Membranes were washed again, developed using the Amersham ECL kit, and then exposed to an X-ray film.

### 2.5. p27^kip1^, Rb Protein, Cleaved Form of Caspase-3, and Phospho-JNK Level Assay

Endothelial cells were seeded at 1.5 × 10^5^ cells per well in 6-well plates, starved overnight with Dulbecco's Modified Eagle Medium (DMEM) medium containing 1% fetal bovine serum (FBS), and incubated with peptide for 30 minutes. The cells were then stimulated with 50 ng/ml VEGF for 0, 2, and 4 hours. After stimulation, cells were lysed in RIPA buffer. The samples were then subjected to SDS-PAGE and western blot analysis with antibodies specific for p27^kip1^, Rb, phospho-JNK, and the cleaved form of caspase-3.

### 2.6. Apoptosis Assay

Apoptosis assays were performed following the manufacturer's instructions. The cells were seeded on cover slips for 12 hours. The cells were starved overnight with media containing a low level of FBS, pretreated with the peptide, and stimulated with VEGF for 4 hours. The cells on the cover slips were fixed in 4% formaldehyde (in PBS) and permeabilized with proteinase K. After equilibration and labeling, the cells were washed and mounted with mounting media and were analyzed under a fluorescence microscope.

### 2.7. Statistical Analysis

The results were shown as mean values ± standard error (mean ± SEM). Statistical analysis was performed using a Student's *t*-test. A level of *p* < 0.05 was considered to be significant.

## 3. Results

### 3.1. AP-Cav Inhibits VEGF-Induced Mouse Colon Endothelial Cell Proliferation

**Figure 1:**
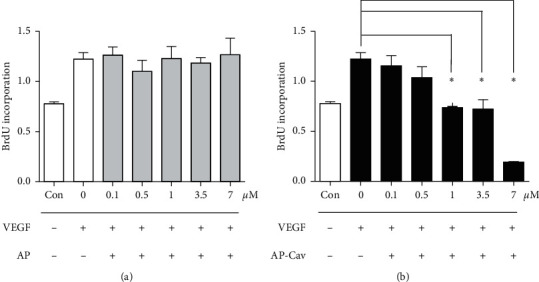
AP-Cav inhibits mouse colon endothelial cell proliferation. (a) AP peptide pretreatment shows no effect on colon endothelial cell proliferation. (b) AP-Cav inhibits endothelial cell proliferation induced by VEGF. Cells were treated with peptide at indicated concentrations for 30 minutes before being treated with VEGF (50 ng/ml) for 4 hours. Data are mean ± s.e.m. from three independent experiments ^*∗*^*p* < 0.05.

An essential component of angiogenic activity is the stimulation of endothelial cell proliferation. As previously shown, overexpression of caveolin-1 protein can inhibit endothelial cell proliferation. However, it is not known whether the AP-Cav peptide similarly inhibits mouse colon endothelial cell proliferation. We determined whether AP-Cav treatment affected cell proliferation by measuring BrdU incorporation as an index of de novo DNA synthesis. Stimulation of mouse colon endothelial cells with 50 ng/ml of VEGF resulted in a 57% increase in BrdU incorporation of control (Figure 1).  Figure 1(a) demonstrates that the AP peptide pretreatment showed no effect on VEGF-induced proliferation. As shown in Figure 1(b), the AP-Cav peptide at the 7 *μ*M concentration showed the most prominent inhibitory effect (decrease of 84% in incorporation compared with VEGF stimulation without the AP-Cav peptide treatment), and at 1 *μ*M and 3.5 *μ*M showed significant inhibition. At 0.1 *μ*M and 0.5 *μ*M concentrations, the AP-Cav peptide treatment shows inhibitory trends. These results suggest that the dose of the AP-Cav dependently inhibits colon endothelial cell proliferation.

### 3.2. AP-Cav Peptide Inhibits VEGF-Induced ERK 1/2 Activity

**Figure 2:**
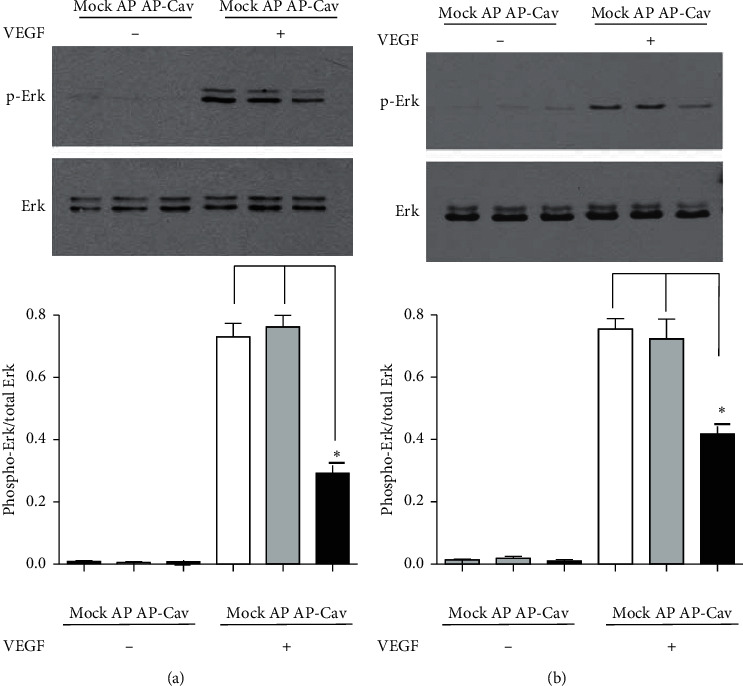
AP-Cav inhibits ERK1/2 phosphorylation. (a) After overnight low serum starvation, the sample was treated for 30 minutes with the AP-Cav peptide and then was stimulated with 50 ng VEGF/ml for 10 minutes. (b) After overnight low serum starvation, 30 minutes AP-Cav peptide treatment, and stimulation with 50 ng VEGF/ml for 2 hours, the ERK1/2 phosphorylation level was determined. Cells were treated with peptide for 30 minutes and then stimulated with 50 ng/ml of VEGF at the indicated times. At the end of the stimulation, cells were immediately lysed in RIPA buffer. The lysates were subjected to SDS-PAGE and western blot analysis with specific antibodies for phospho-ERK 1/2 and total ERK 1/2. Bar graph represents the phospho-ERK 1/2 to total ERK 1/2 ratio normalized to beta-actin levels. The data are mean ± s.e.m. from three independent experiments, ^*∗*^*p* < 0.05.

VEGF stimulation of endothelial cells results in activation of ERK 1/2 which facilitates proliferation responses. A previous study demonstrated that overexpression of caveolin-1 protein inhibits ERK 1/2 activity [8]. However, it is unknown if the AP-Cav peptide can similarly inhibit ERK 1/2 activity in microvascular colon endothelial cells. The effect of AP-Cav treatment on VEGF-induced mitogenic signaling was determined by measuring the degree of phosphorylation of ERK 1/2 by western blot analysis.  Figure 2 shows that the AP control peptide or mock treatment plus 50 ng/ml of VEGF results in phosphorylation of ERK 1/2 within 10 minutes. At 2 hours after stimulation, the ERK 1/2 activity still exists. Interestingly, pretreatment with the AP-Cav peptide before the addition of VEGF resulted in a decrease of phospho-ERK at the 10-minute and 2-hour time points. These data demonstrate that the AP-Cav peptide prevents VEGF-dependent ERK 1/2 phosphorylation, which is involved in endothelial cell proliferation.

### 3.3. AP-Cav Peptides Downregulate p27^kip1^ and Rb Levels

**Figure 3:**
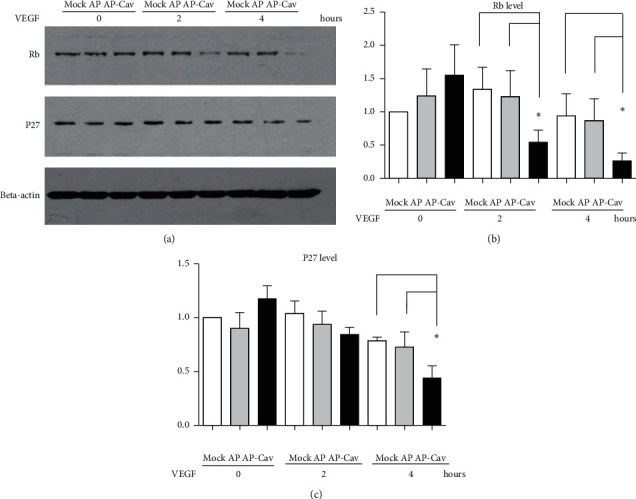
Effect of AP-Cav on cell cycle protein level of p27^kip1^, Rb in mouse colon endothelial cells. (a) After overnight low serum starvation, 30 minute AP-Cav peptide treatment, and stimulation with 50 ng VEGF/ml for 0, 2, and 4 hours, cells were subjected to SDS-PAGE and western blot analysis with specific antibodies for p27^kip1^ or Rb. Beta-actin was used as a loading control. (b) Bar graph represents relative Rb level normalized to beta-actin level. (c) Bar graph represents relative level of p27^kip1^ normalized to beta-actin level. Data are mean ± s.e.m. from three independent experiments, ^*∗*^*p* < 0.05.

We analyzed the effect of AP-Cav on the levels of p27^kip1^. The 27^kip1^ is a cyclin-dependent kinase (CDK) inhibitor that leads to G1 arrest through inhibiting the activity of G1 cyclin-cdks. Mitogenic signals lead to phosphorylation of p27^kip1^, and the phosphorylated p27^kip1^ is degraded by the ubiquitin pathway [9]. As shown in Figure 3, without VEGF stimulation, the level of p27^kip1^ was slightly higher after 30 minutes of AP-Cav peptide treatment, compared with the AP peptide treatment; however, after VEGF stimulation for 2 hours and 4 hours, the p27^kip1^ level was lower than the control treatment. These results suggest that the AP-Cav peptide treatment downregulates p27^kip1^ levels when the endothelial cells are stimulated with VEGF.

Next, we analyzed the effect of AP-Cav on the level of the tumor suppressor, Rb, which is believed to play an important role in the control of cell proliferation. Rb protein binds to the transcription factor E2F, and this reaction leads to downregulation of the transcription of genes required to pass through the restriction point in the late G1 phase [10]. It is also shown that Rb protein plays a role in preventing cells from undergoing apoptosis [11, 12]. To determine whether the AP-Cav peptide affects the Rb protein level, mouse colon endothelial cells were pretreated for 30 minutes with AP-Cav or the AP peptide, and then stimulated with VEGF as described above. Western blot analysis showed that Rb protein levels are downregulated with the AP-Cav peptide treatment when compared with control cells (Figure 3). At 4 hours, Rb protein levels were dramatically downregulated. The level of Rb protein was not upregulated, suggesting Rb downregulation may lead to endothelial cell apoptosis.

**Figure 4:**
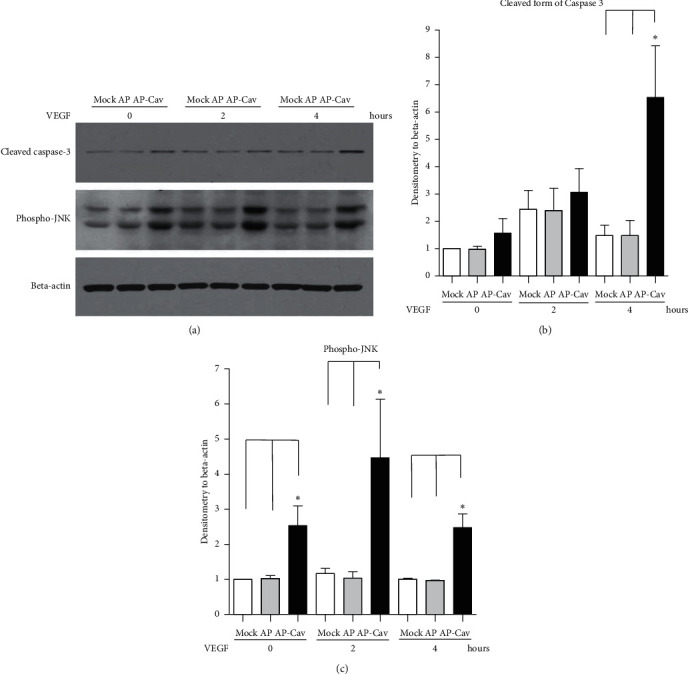
AP-Cav peptide upregulates caspase-3 activity and JNK activity in mouse colon endothelial cells. (a) Mouse colon endothelial cells were starved in media containing 1% FBS overnight, treated with the peptide for 30 minutes, and stimulated with 50 ng/ml of VEGF for 0, 2, and 4 hours. At the end of the stimulation, cells were lysed in boiling SDS-sample buffer, and cell lysates were subjected to SDS-PAGE and western blot analysis with specific antibodies for the activated form of caspase-3 and phospho-JNK. The protein loading was probed with antibody for beta-actin. (b) Bar graph represents relative cleaved form of caspase-3 normalized to beta-actin level. (c) Bar graph represents phospho-JNK level normalized to beta-actin level. Data are mean ± s.e.m. from three independent experiments, ^*∗*^*p* < 0.05.

AP-Cav upregulates caspase-3 and JNK activity and induces apoptosis. Since Rb protein has been shown play a role in apoptosis [11] and is downregulated by AP-Cav treatment in mouse colon endothelial cells (Figure 3), we then check whether AP-Cav regulates apoptosis in colonic endothelial cells. Caspase-3 is one of the key enzymes of apoptosis, as it is either partially or completely responsible for the proteolytic cleavage of many key proteins involved in apoptosis [13]. Activation of caspase-3 requires digestion of the inactive caspase-3 zymogen into activated p17 and p12 fragments [14]. To test the hypothesis that the AP-Cav peptide inhibition of endothelial cell proliferation may result from apoptosis, mouse colon endothelial cells were stimulated with 50 ng/ml VEGF after 30 minutes of the AP-Cav peptide pretreatment. The cell lysate was collected at 0, 2, and 4 hours. Western blot analysis was used to assay the caspase-3 activity using an antibody that only recognizes activated caspase-3. As shown in Figure 4, after endothelial cells were starved in DMEM medium containing 1% FBS, the pretreatment of AP-Cav leads to upregulated caspase-3 activity compared to the AP peptide treatment with and without VEGF stimulation.

JNK 1/2 are stress-activated protein kinases and play an important role in inflammation and apoptosis [15]. Thus, we detected the JNK 1/2 activity with western blot analysis. As shown in Figure 4, JNK 1/2 activity greatly increased after a 30-minute treatment with AP-Cav peptide alone compared with the control and AP peptide-treated cells. After 2 hours and 4 hours of VEGF stimulation, JNK 1/2 activity in the AP-Cav treated cells was still higher than the control and AP peptide-treated cells.

**Figure 5:**
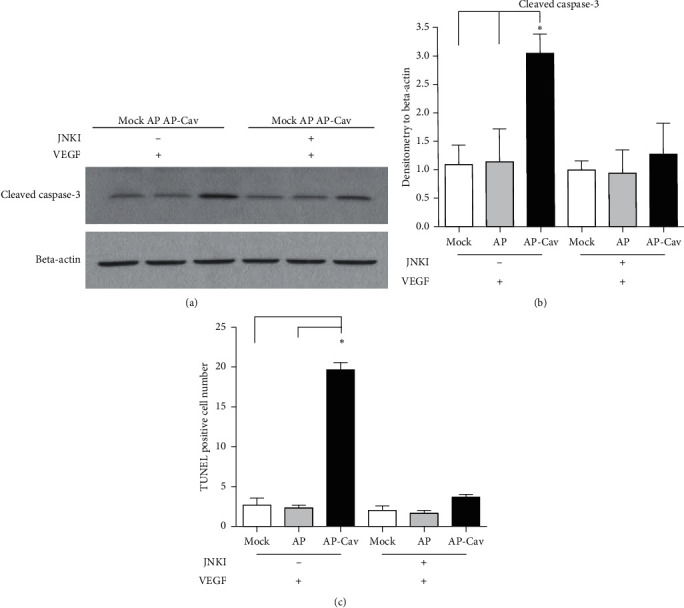
JNK inhibition reverses the effect of AP-Cav on mouse colon endothelial cell apoptosis. Mouse colon endothelial cells were starved in media containing 1% FBS overnight, treated with JNK inhibitor for 1 hour, incubated for 30 minutes with AP or AP-Cav peptide, and stimulated with 50 ng/ml of VEGF for 4 hours. (a) At the end of the treatment, the cells were lysed in boiling SDS-sample buffer followed by western blot analysis with antibody for activated form of caspase-3. (b) Bar graph represents relative cleaved form of caspase-3 level normalized to beta-actin level. (c) At the end of the treatment, the cells were fixed and analyzed using fluorescein FragEL DNA fragmentation detection kit and then evaluated for apoptotic cells under a fluorescence microscope. The TUNEL positive cell number per field under 10x objective was counted. Data are mean ± s.e.m. from three independent experiments, ^*∗*^*p* < 0.05.

As caspase-3 and JNK 1/2 play central roles during cell apoptosis, we detected whether caspase-3 activity is regulated by JNK1/2 in colon endothelial cells. The western blot analysis (Figures 5(a) and 5(b)) showed that JNK inhibition decreased levels of cleaved caspase-3, which further suggests that AP-Cav might induce endothelial apoptosis through JNK signaling pathway. This was verified as shown in Figure 5(c); after starvation in low serum medium, more than 5 times as many endothelial cells underwent apoptosis after 4 hours of VEGF stimulation compared with cells treated with the AP peptide. As expected, pretreatment of cells with JNK inhibitors prevents AP-Cav-induced apoptosis (Figure 5(c)).

## 4. Discussion

Angiogenesis has recently been shown to play a role in the perpetuation and enhancement of colitis, which is stimulated by inflammatory factors such as VEGF. Since endothelial cell proliferation is the primary step of angiogenesis, it is an attractive target for colitis treatment through antiangiogenenesis. A previous report that overexpression of caveolin-1 protein inhibits endothelial cell proliferation suggests that caveolin-1 protein might be selected as a target to treat angiogenesis-related disease [8]. Consistently, AP-Cav peptide treatment alleviates DSS-induced colitis in mice and inhibits colon angiogenesis [1]. Here, we report that as an important domain of caveolin-1 protein, AP-Cav peptide inhibits mouse colon endothelial cell proliferation, and induces mouse colon endothelial cell apoptosis.

As a major component of caveolae, caveolin-1 seems to play a dual role such as blocking the basal activity of enzymes residing in caveolae and facilitating their activation upon agonist stimulation [16, 17]. Caveolin-1 has been shown to coimmunopreciptate with VEGFR2 in caveolae and thereby regulate VEGF signaling [4]. One possible mechanism by which AP-Cav acts on VEGF signaling might be through disruption of the binding of caveolin-1 and signaling molecules to CSD binding sites and preventing the signaling molecules from locating to the caveolae, thus preventing signal transduction, such as through the VEGF-ERK 1/2 pathway. AP-Cav is also shown to inhibit NGF-induced ERK 1/2 phosphorylation in oligodendroglial cells [18], ERK 1/2 phosphorylation in human lung fibroblasts, and VEGF-induced ERK 1/2 phosphorylation in ovine fetoplacental artery endothelial (oFPAE) cells. Interestingly, there is a difference between the effects of the CSD on endothelial cell signal transduction when compared with the whole caveolin-1 molecule. One difference is that overexpression of the whole caveolin-1 protein inhibits Rb protein phosphorylation [8], while the AP-Cav peptide treatment lowers Rb protein level. This difference might be due to the fact that the whole caveolin-1 protein is composed of 178 amino acids, while AP-Cav contains only part of the caveolin-1 protein and is composed of 36 amino acids. Interestingly, Rb role in apoptosis is still controversial, and Rb can either inhibit or promote apoptosis depending on the cellular context [19]. Our study demonstrated that AP-Cav inhibits Rb protein expression in colon endothelial cells. It will be interesting to investigate the role of the Rb/E2F pathway in colon endothelial cell apoptosis.

Our experiments show that AP-Cav promotes JNK 1/2 activation in mouse colon endothelial cells. However, other researchers have shown that AP-Cav inhibits JNK phosphorylation in human lung fibroblasts and has a remarkable ability to inhibit bleomycin-induced epithelial cell apoptosis [20]. It is also been shown that AP-Cav inhibits JNK activity in oFPAE cells [21]. This difference might due to AP-Cav playing a different role in different cellular contexts. It has also been shown that JNK 1 and JNK 2 protein levels and phospho-JNK are increased in inflamed colonic mucosa of patients with IBD [22]. However, in the DSS model of experimental colitis, knocking out JNK 1 and JNK 2 does not prevent mice from developing chronic colitis, and JNK 2 deletion actually aggravates DSS-induced colitis [23]. Therefore, further research is necessary to conclude whether JNK 1/2 activation plays a protective role in colitis. If JNK 1/2 activation plays a harmful role in colitis, then AP-Cav needs to be dosed concurrently with JNK 1/2-specific inhibitors to treat colitis. Scanning the protein amino acid sequence of JNK 1/2 did not reveal the caveolin-1-binding motif in JNK 1/2, suggesting that JNK 1/2 might not be localized to the caveolae and that AP-Cav might indirectly activate JNK 1/2 through other molecules. Consistent with our finding, it has been reported that AP-Cav can induce JNK activation in N9 microglia and BMDM cells [24]. Furthermore, our finding that AP-Cav induces mouse colon endothelial cell apoptosis is consistent with numerous studies that show that caveolin-1 acts as a proapoptotic protein in NIH-3T3 fibroblasts [25], T24 bladder carcinoma cells, MEFs [26], human 293 cell lines, and Hela cells [27].

We found that in mouse colon endothelial cells, VEGF-induced activation of ERK 1/2 was suppressed by AP-Cav treatment. Our results show that the AP-Cav peptide pretreatment downregulates Rb protein expression and upregulates caspase-3 activity, inducing endothelial cell apoptosis. Although our experiments indicated AP-Cav-regulated colon endothelial cell survival through JNK and ERK 1/2 pathways, further research is necessary to conclude whether those *in vitro* occurrences were due to the mechanism of AP-Cav's inhibition of DSS-induced colitis *in vivo*.

## Data Availability

All the data used in the manuscript is in the manuscript submitted.

## Abbreviations

AP: Antennapedia
AP-Cav: Antennapedia-conjugated caveolin-1-scaffolding domain
VEGF: Vascular endothelial growth factor
BrdU: Bromodeoxyuridine
ERK1/2: Extracellular signal-regulated kinase 1/2
JNK: c-Jun *N*-terminal kinase
Rb: Retinoblastoma
MAP: Mitogen activated protein
PDGF: Platelet-derived growth factor
CSD: Caveolin-1-scaffolding domain
PVDF: Polyvinylidene difluoride
DMEM: Dulbecco's modified eagle medium
FBS: Fetal bovine serum
oFPAE: Ovine fetoplacental artery endothelial.
